# Nitrogen Deprivation Drives Red Motile Cell Formation in *Haematococcus pluvialis*: Physiological and Transcriptomic Insights

**DOI:** 10.3390/metabo15060388

**Published:** 2025-06-10

**Authors:** Hailiang Xing, Na Zhou, Kai Liu, Xiaotian Yan, Wanxia Li, Xue Sun, Liuquan Zhang, Fengjie Liu, Nianjun Xu, Chaoyang Hu

**Affiliations:** 1 Key Laboratory of Marine Biotechnology of Zhejiang Province, School of Marine Sciences, Ningbo University, Ningbo 315832, China; 2101130075@nbu.edu.cn (H.X.);; 2 Grantham Institute for Climate Change and the Environment, Imperial College London, London SW7 2AZ, UK

**Keywords:** astaxanthin, fatty acids, photosynthesis, lipids, amino acids, transcriptomics

## Abstract

**Background:** Natural astaxanthin, a commercially valuable carotenoid, is primarily sourced from *Haematococcus pluvialis*, a microalga known for its remarkable resilience to environmental stress. **Methods:** In this study, the physiological and transcriptomic responses of *H. pluvialis* to ND were investigated at various time points under high light conditions. **Results:** Under high light conditions, nitrogen deprivation (ND) enhances astaxanthin content (33.23 mg g^−1^) while inhibiting the formation of the secondary cell wall (SCW), increasing astaxanthin content by 29% compared to the nitrogen-replete group (25.64 mg g^−1^); however, the underlying mechanisms remain unclear. ND reduced chlorophyll fluorescence parameters, elevated reactive oxygen species (ROS) levels, and increased starch and total sugar accumulation while decreasing protein and lipid content. Fatty acid content increased on the first day but had declined by the fifth day. A transcriptomic analysis revealed substantial alterations in gene expression in response to ND. Genes associated with the TCA cycle, glycolysis, astaxanthin biosynthesis, and cell motility were upregulated, while those involved in photosynthesis, lipid synthesis, ribosome biogenesis, amino acid synthesis, and SCW synthesis were downregulated. Additionally, ND modulated the expression of genes involved in ROS scavenging. **Conclusions:** These findings provide critical insights into the adaptive mechanisms of *H. pluvialis* in response to ND under high light, contributing to the development of strategies for enhanced production of astaxanthin-rich motile cells.

## 1. Introduction

Astaxanthin, a high-value carotenoid with exceptional antioxidant properties, is extensively utilized in animal feed, cosmetics, functional foods, and pharmaceuticals [1,2]. Its sources are primarily categorized into chemical synthesis and natural extraction. Although chemically synthesized astaxanthin is more cost-effective, it exhibits lower bioactivity than its naturally derived counterpart and is unsuitable for use in functional foods and cosmetics due to potential food safety concerns [3,4,5]. Natural sources of astaxanthin include the exoskeletons of shrimp and crabs, flamingo feathers, the yeast *Phaffia rhodozyma*, and specific microalgae species such as *Haematococcus pluvialis*, *Chromochloris zofingiensis*, *Microcystis aeruginosa*, and *Oedocladium carolinianum* [6]. Among these, *H. pluvialis*, a unicellular freshwater microalga commonly found in aquatic ecosystems, is considered the optimal source for commercial production, as its astaxanthin content can exceed 5% of its dry cell weight [6,7].

The production of natural astaxanthin from *H. pluvialis* typically employs a two-phase cultivation strategy. In the first stage, cells are cultivated under optimal environmental conditions (light, temperature, pH, and nutrient supply) to promote rapid biomass accumulation. Subsequently, in the second stage, specific abiotic stresses (e.g., high light and nutrient deprivation) are employed to stimulate astaxanthin synthesis [4,7,8]. “High light” is a straightforward and effective strategy for enhancing astaxanthin production by *H. pluvialis* [9,10,11], which was proven in our previous studies [12]. However, under these cultivation conditions, *H. pluvialis* often tends to develop a rigid, multilayered secondary cell wall (SCW). Although this structure enhances the cell’s resistance to environmental stress, it significantly hinders astaxanthin extraction and reduces its bioavailability [13,14,15]. When these cells, with intact cell walls, are used as feed, fish exhibit inferior growth performance and pigmentation compared to those fed extracted astaxanthin directly [16,17]. Recently, a strategy has been proposed to cultivate motile cells rich in astaxanthin, which could improve bioavailability and simplify extraction processes [18,19,20].

Nitrogen plays a vital role in plant growth and development, as it is a fundamental building block of amino acids, proteins, nucleic acids, and crucial components of cell walls and membranes [21]. Nitrogen deprivation (ND) disrupts the metabolic network, affecting the acquisition, transport, and assimilation of nitrogenous nutrients in plant tissues, thereby influencing the growth and developmental processes of plants [22]. ND can also reduce the photosynthetic capacity of plants and lead to the degradation of proteins, chlorophyll, and lipids [23,24,25]. It has been demonstrated that nitrogen levels significantly affect the structure and composition of plant cell walls. For instance, ND has been shown to decrease the cellulose content while simultaneously increasing the lignin content in grape cell walls [26]. Additionally, ND has been found to modify the lignin profile and enhance the accumulation of hemicellulose glucans and xylans in sorghum seedlings [27]. ND has been extensively established as an effective approach to stimulating astaxanthin biosynthesis in *H. pluvialis* under high light, with significant progress documented in recent studies [28,29,30,31,32]. While previous studies were primarily centered on elucidating the regulatory mechanisms of carotenoid metabolism, comprehensive studies systematically investigating ND-induced morphological transformations in algal cells remain notably limited. Our preliminary work has demonstrated that ND can induce the emergence of red motile cells under high light exposure [19], yet the molecular underpinnings governing this morphological shift persist as a critical knowledge gap. Although two research groups have observed analogous cellular transformations [33,34], two notable research gaps remain unaddressed: (1) a lack dynamic physiological monitoring to resolve the spatiotemporal progression of red motile cell development, and (2) insufficient transcriptomic investigations to delineate the nitrogen-responsive signaling networks orchestrating this process. These mechanistic uncertainties fundamentally constrain our ability to address a pivotal question in nitrogen metabolism research: How does nitrogen deprivation mediate the coordinated regulation of biosynthetic carbon partitioning between astaxanthin and SCW components?

In this study, a comprehensive analysis was conducted on the effects of ND on *H. pluvialis* under high light conditions at three time points, focusing on cellular morphology, photosynthetic parameters, physiological and biochemical responses, and reactive oxygen species (ROS) generation. Additionally, a transcriptomic analysis was conducted at two time points to examine the transcriptional responses of *H. pluvialis* to ND under high light exposure. The findings advance the understanding of the adaptive mechanisms employed by *H. pluvialis* in response to ND under high light exposure, offering valuable theoretical support for its potential applications in industry.

## 2. Materials and Methods

### 2.1. Algal Growth and Processing

In this study, *H. pluvialis* NBU489 was sourced from the Algal Culture Collection at Ningbo University (Ningbo, China). A two-stage cultivation strategy was implemented based on established protocols [19]. In the initial phase, algal cells were cultured in a 1 L bubble photobioreactor filled with 800 mL of sterilized NUB^3#^ medium. The medium contained the following nutrients: 0.1 g/L NaNO_3_, 0.02 g/L Na_2_EDTA, 0.01 g/L K_2_HPO_4_, 2.5 × 10^−3^ g/L FeSO_4_·7H_2_O, 2.5 × 10^−4^ g/L MnSO_4_, 5 × 10^−9^ g/L VB_1_ (thiamine), and 5 × 10^−10^ g/L VB_12_ (cyanocobalamin). Cultures were maintained under a 12:12 h light–dark cycle at a surface photosynthetic photon flux density (PPFD) of 30 μmol photons·m^−2^·s^−1^, and a constant temperature of 25 ± 1 °C for 13 days. At the end of this phase, cells in the stationary growth phase were collected by centrifugation at 3000× *g* for 5 min. The harvested biomass was resuspended in 50 mL of either nitrogen-replete or nitrogen-deprived medium [19], prepared in 100 mL conical flasks. These were designated as the nitrogen-deprived (ND) group and nitrogen-replete (NR) group, respectively. The initial cell concentration was approximately 2.30 × 10^5^ cells/mL. To induce astaxanthin accumulation, cultures were subsequently exposed to high light intensity (PPFD of 200 μmol·m^−2^·s^−1^) under continuous illumination at 25 ± 1 °C for 10 days. Each group consisted of three replicates. Algal cells were harvested on days 1, 5, and 10 for comprehensive morphological observations and analyses of chlorophyll fluorescence parameters, total sugars, proteins, starch, lipids, and free fatty acid contents.

### 2.2. Morphological Observation of Algal Cells

To examine algal cell morphology, 1 mL of the culture was pelleted by centrifugation at 1000× *g* for 30 s. In total, 10 μL of the concentrated algal culture was placed on a glass slide and observed using an optical microscope equipped with a digital camera (ToupCam™, Touptek, Hangzhou, China).

### 2.3. Determination of Astaxanthin Content

The quantification of astaxanthin was performed using a modified version of the method described by Li et al. [35]. Briefly, 5 mg (DW) of freeze-dried algal powder was mixed with 10 mL (V) of DMSO and incubated in a 65 °C water bath for 15 min. The mixture was then centrifuged at 3000× *g* for 5 min, and the supernatant was transferred to a clean centrifuge tube. A second 10 mL aliquot of DMSO was added to the pellet, followed by a repetition of the incubation and centrifugation steps. The red-colored supernatants were then combined, and the absorbance of the astaxanthin extract was measured at 530 nm (A_530_) using a spectrophotometer.

A standard astaxanthin solution (≥98% UV purity; Solarbio, Beijing, China) was used to generate a calibration curve. Five concentrations of astaxanthin (13.8, 6.9, 3.45, 1.725, and 0.8625 μg/mL in DMSO) were prepared and their absorbance was measured at 530 nm. The resulting standard curve was represented by the equation y (mg/L) = 9.2961 × A_530_ + 0.074, with an R^2^ value of 0.9996, where A_530_ represents the absorbance at 530 nm. The astaxanthin content (C_Astaxanthin_, mg/g) was calculated using the following formula: C_Astaxanthin_ = (9.2961 × A530 + 0.074) × V/DW.

### 2.4. Chlorophyll Fluorescence Analysis

Chlorophyll fluorescence were conducted using a PSI AquaPen-C fluorometer (Photon System Instruments, Drásov, Czech Republic), following the protocol established by Li [36]. The assessed parameters included the maximum quantum efficiency of PSII (Fv/Fm), the effective quantum yield (Fv′/Fm′), non-photochemical quenching (NPQ), and the relative electron transport rate (rETR).

### 2.5. Analysis of Total Sugar Content

The intracellular total sugar content was quantified using a commercial assay kit (Suzhou Comin Biotechnology Co., Ltd., Suzhou, China). Briefly, 10 mL of algal culture was centrifuged to harvest the cells, which were then transferred to a 2 mL micro-grinding tube. Grinding beads were added, and the cells were disrupted using a tissue grinder at 60 Hz for 2 min. Following cell disruption, total sugar content was determined according to the manufacturer’s instructions.

### 2.6. Quantification of Protein Content

The protein content of *H. pluvialis* was determined following a previously described method [37]. Briefly, a 10 mL algae suspension was centrifuged, and the resulting pellet was transferred to a 2 mL grinding tube for homogenization. Subsequently, 200 µL of 1 M NaOH was added, followed by incubation in a water bath at 80 °C for 10 min. Next, 800 µL of distilled water was added and thoroughly mixed by vortexing. The sample was centrifuged at 12,000× *g* for 30 min, and the supernatant was collected into a 5 mL centrifuge tube. The extraction process was repeated twice, and all supernatants were pooled and diluted to a final volume of 10 mL. A 2 mL aliquot of the extract or protein standard was mixed with 5 mL of Coomassie Brilliant Blue reagent and incubated at room temperature for 15 min. Next, 200 µL of the mixture was transferred to a 96-well microplate, and the absorbance was measured at 595 nm using a microplate reader (Molecular Devices, Silicon Valley, San Jose, CA, USA). Bovine serum albumin (Shanghai Macklin Biochemical Co., Ltd., Shanghai, China) served as the calibration standard, with seven concentration gradients (0 to 60 mg/L) for the standard curve. The standard curve equation was Y (mg/L) = 151.6X − 3.9847, where Y is the protein concentration and X is the absorbance at 595 nm (R^2^ = 0.9985).

### 2.7. Quantification of Starch Content

The starch content of *H. pluvialis* was determined employing a commercial starch assay kit (Suzhou Comin Biotechnology Co., Ltd., Suzhou, China). Algal biomass was harvested by centrifuging 10 mL of the suspension, followed by transferring the pelleted cells into a 2 mL grinding vessel. Ceramic grinding beads were introduced, and cellular disruption was performed using a mechanical homogenizer operated at 60 Hz for 2 min. Starch quantification was then carried out strictly according to the manufacturer’s protocol, ensuring standardized measurement conditions.

### 2.8. Quantification of Lipid Content

The lipid content of *H. pluvialis* was determined via a sequential solvent extraction procedure. Freeze-dried algal biomass (30 mg, recorded as M) was mechanically disrupted in a 2 mL grinding tube. A binary solvent system of chloroform–methanol (2:1 *v*/*v*; 1 mL) was added to the homogenate, followed by vigorous agitation (10 min, 25 °C). Phase separation was achieved by centrifugation (12,000× *g*, 5 min), and the resulting supernatant was quantitatively transferred to a pre-weighed aluminum foil dish (M1). The remaining biomass underwent iterative extraction cycles (2–3 repetitions) with fresh solvent aliquots until complete pigment elimination, as evidenced by a visually homogeneous grayish-white reside. The combined organic extracts were evaporated to a constant weight in a precision-controlled drying oven (37 ± 0.5 °C). Gravimetric analysis provided the total lipid mass (M2), based on which the lipid percentage was computed as: Lipid content (%) = [(M2 − M1)/M] × 100.

### 2.9. Quantification of Free Fatty Acids Composition

A 0.01 g sample of lyophilized algae was finely ground and extracted with 1 mL of a chloroform–methanol mixture (with a volume ratio of 2:1), by shaking for 10 min. After centrifugation, the supernatant was collected and transferred into a 15 mL centrifuge tube. The residue was re-extracted 2–3 times with 1 mL portions of the same solvent until the algal biomass turned grayish-white. All supernatants were pooled and evaporated to dryness. Next, 2 mL of methanol–sulfuric acid solution (with a volume ratio of 5:95) was added to the dried extract and incubated in a 70 °C water bath for 4 h. This derivatization step converted the fatty acids into their corresponding methyl esters. Subsequently, 2 mL each of *n*-hexane and ultrapure water were added to the reaction mixture. After vigorous vortex mixing, the biphasic system was allowed to stand for 30 min to enable phase separation. The resultant hydrophobic phase was collected via 0.22 μm PTFE membrane filtration prior to chromatographic analysis using an Agilent 8860 GC coupled with a 5977C MSD mass spectrometer. Detailed analytical procedures were described in our previous study [37].

### 2.10. Qualitative and Quantitative Analysis of Reactive Oxygen Species

Algal cells subjected to various treatments were harvested at 0, 12, 24, and 48 h for ROS analysis. For each sample, 2 mL of algae culture was centrifuged at 3000× *g* for 5 min. The supernatant was discarded, and the pellet was washed 2–3 times with 0.05 M PBS (pH 7.0). The pellet was then resuspended in 1 mL of 10 μM H2DCFH-DA (Solarbio, Beijing, China) and incubated at 37 °C in the dark for 30 min. After incubation, the cells were centrifuged at 3000× *g* for 5 min and the supernatant was discarded. The pellet was washed three times with 0.05 M PBS, then resuspended in 1 mL of PBS. Subsequently, 200 μL of the suspension was transferred to a black 96-well microplate. Fluorescence signals of intracellular ROS were detected using a multifunctional fluorescence microplate reader (Molecular Devices, San Jose, CA, USA) and observed with an inverted fluorescence microscope (Nikon, Tokyo, Japan).

### 2.11. Transcriptome Analysis

Total RNA was extracted from algal cells on day 1 from both ND and NR groups for RNA sequencing analysis. The procedures for cDNA library construction, RNA sequencing, sequence assembly, gene annotation, expression quantification, differential gene expressed analysis (DEGs), and GO/KEGG enrichment analysis were performed as described in our previous studies [8,10]. The raw RNA sequencing data supporting this study have been deposited in the NCBI Sequence Read Archive under the accession number PRJNA1256639.

### 2.12. Validation of DEGs by qRT-PCR

To validate the DEGs identified from transcriptome analysis, several genes involved in fatty acid biosynthesis, astaxanthin biosynthesis, and mannose metabolism were randomly selected for qRT-PCR. Gene-specific primers were designed using Primer Premier 5 software (Premier Biosoft International, San Francisco, CA, USA) and are listed in Table S1. We used 18S rRNA as the internal reference gene, and relative gene expression levels were calculated using the 2^−ΔΔCt^ method.

### 2.13. Statistical Analysis

Each experiment was conducted with three to five biological replicates, and the results were presented as means ± standard deviation. Differences in ROS level, chlorophyll fluorescence parameters, sugar content, protein content, starch content, lipid content, and free fatty acid content between ND and NR groups at the same timepoints were analyzed using an Independent-Samples *t*-test in SPSS 22.0. Statistical significance was defined as *p* < 0.05 (indicated by *), and extremely significant difference was defined as *p* < 0.01 (indicated by **). Figures were constructed and edited using Origin 2024 and Adobe Illustrator 2020.

## 3. Results

### 3.1. Effects of Nitrogen Deprivation on Morphology

**Figure 1:**
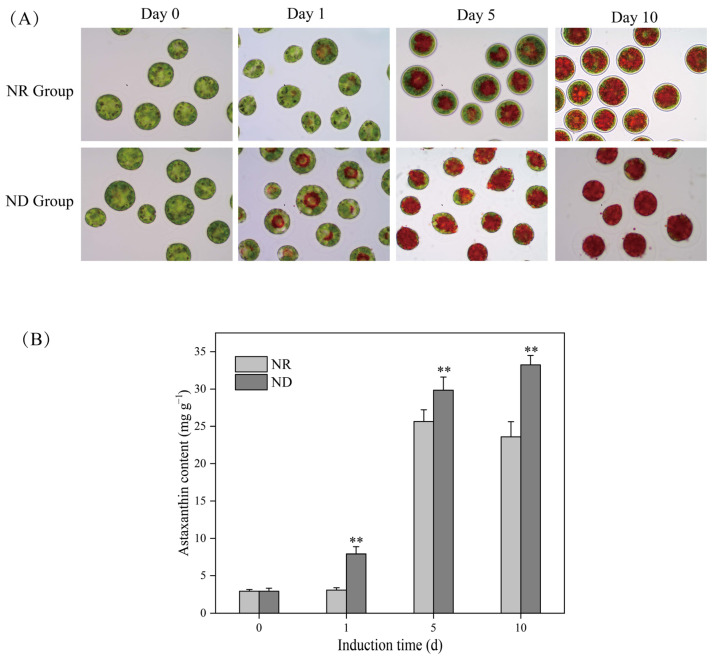
Light microscopy images (**A**) and astaxanthin content (**B**) of *H. pluvialis* cells at various time points under nitrate-replete (NR) and nitrate-deprived (ND) conditions. ** indicates highly significant (*p* < 0.01) differences between the NR and ND groups for the corresponding index at the same time point.

The morphology of the algal cells in the nitrogen-replete (NR) group underwent significant changes from day 0 to day 10 post-exposure to high light, which can be summarized in the following key aspects: firstly, the shedding of the flagella led to the loss of the motility of the algal cells; secondly, the gradual formation of a secondary cell wall (SCW) transformed the originally thin-walled and oval vegetative cells into thick-walled and spherical cyst cells; and lastly, the accumulation of astaxanthin caused the color of the algal cells to shift from green to red (Figure 1). This result is consistent with our previous studies [8,10,11,18,19,20,38]. However, for the algal cells in the nitrogen-deprived (ND) group, apart from a gradual shift in color from green to red, there were no significant changes observed in the cell wall (Figure 1), which is consistent with the morphologic changes observed in the ND-treated cells in previous studies [19,33,34]. The cells in the ND group were redder than those in the NR group at the same time point after day 1, corresponding to accelerated astaxanthin accumulation, as confirmed by quantitative analysis (Figure 1B). From day 1 onward, the astaxanthin content in the ND group was markedly elevated compared to that in the NR group. On days 5 and 10, the astaxanthin levels in the ND group reached 26.85 mg g^−1^ and 33.18 mg g^−1^, respectively. These values represent increases of 1.73 and 1.29 times the corresponding values in the NR group, which were 15.48 mg g^−1^ and 25.64 mg g^−1^, respectively (Figure 1B).

### 3.2. Effects of Nitrogen Deprivation on ROS Levels

**Figure 2:**
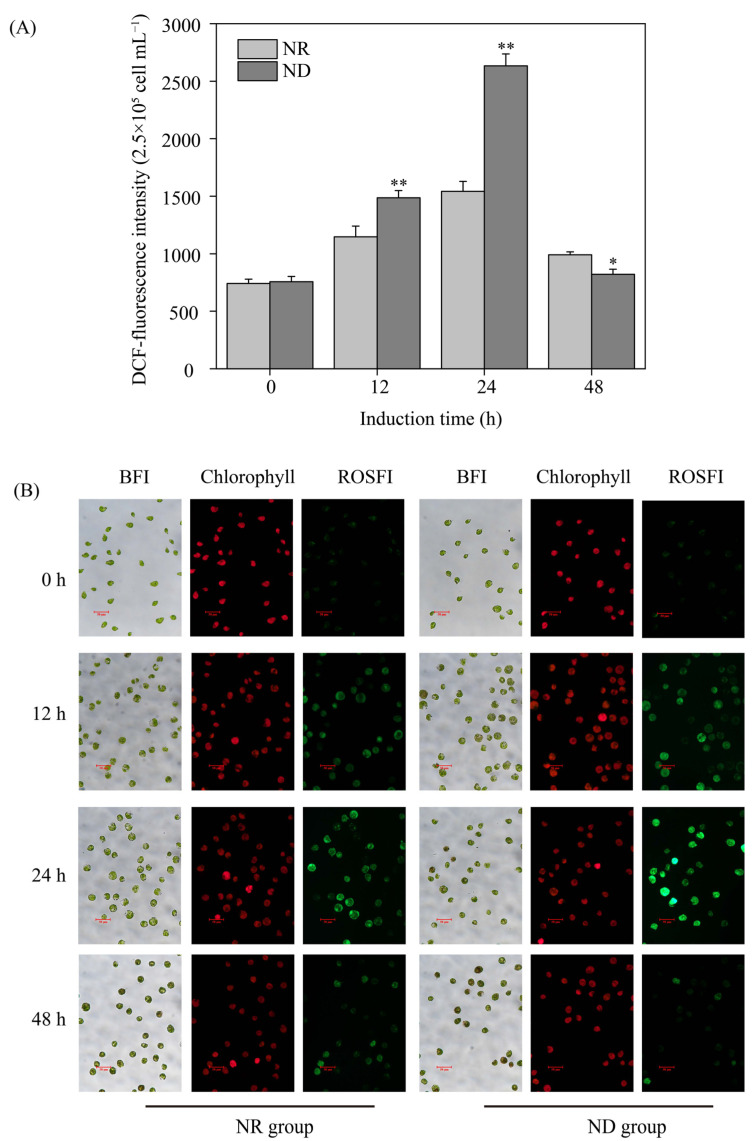
Comparison of reactive oxygen species (ROS) content (**A**) and the bright field images (BFI), chlorophyll images and ROS fluorescent images (ROSFI) (**B**) of *H. pluvialis* cells between NR and ND groups at different time points. * and ** indicate significant (*p* < 0.05) and highly significant (*p* < 0.01) differences, respectively, between the NR and ND groups for the corresponding index at the same time point.

Various environmental factors, such as high light and ND, induce oxidative stress, resulting in the generation of reactive oxygen species (ROS) within plant cells, including algae [39]. In this study, the ROS fluorescence intensity and levels in both the ND and NR groups exhibited a pattern of initially increasing and then decreasing within the first 48 h of high light exposure, peaking at 24 h (Figure 2). The ROS fluorescence intensity in the ND group was about 50% higher than that in the NR group at 12 and 24 h after high light exposure. However, at 48 h, the ND group exhibited markedly reduced ROS level compared to the NR group (Figure 2). These findings suggest that ND induces a sharp increase in ROS levels in *H. pluvialis* without triggering cell death.

### 3.3. Effects of Nitrogen Deprivation on Photosynthetic Parameters

**Figure 3:**
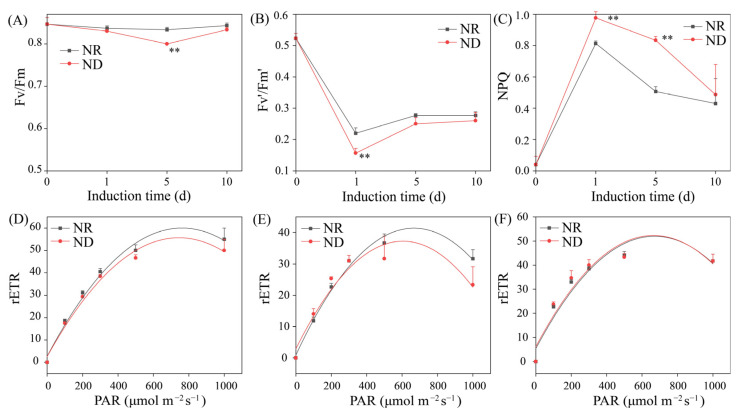
Comparison of chlorophyll fluorescence parameters between NR and ND groups. (**A**) Fv′/Fm′; (**B**) Fv/Fm; (**C**) NPQ; (**D**–**F**) rETR. ** indicates highly significant (*p* < 0.01) differences between the NR and ND groups for the corresponding index at the same time point.

ND affects photosynthesis and chlorophyll fluorescence in higher plants [40]. Therefore, the impact of ND on the chlorophyll fluorescence parameters of *H. pluvialis* under high light conditions was investigated. The maximum photochemical efficiency of photosystem II (Fv/Fm) signifies the system’s ability to harness light energy at its peak intensity. Under high light conditions, the Fv/Fm ratio in the NR group remained relatively stable throughout the induction period. Conversely, the Fv/Fm ratio in the ND group declined from day 0 to day 5, followed by a gradual increase. On day 5, the ND group displayed a significantly lower Fv/Fm ratio compared to its NR counterpart (Figure 3A). The actual light energy conversion efficiency under cultivation conditions, denoted as Fv′/Fm′, reflects the activity of photosynthesis [41]. Both groups experienced a rapid decrease in Fv′/Fm′ from day 0 to day 1, followed by a gradual increase toward stabilization. On day 1, the ND group exhibited a significantly lower Fv′/Fm′ ratio compared to the NR group, a pattern that persisted through days 5 and 10, although the subsequent differences were not statistically significant (Figure 3B). These findings suggest that ND suppresses the photosynthetic activity of algal cells, particularly during the early cultivation phase.

Non-photochemical quenching (NPQ) is an essential photosynthetic mechanism that dissipates excess absorbed light energy as heat, thereby safeguarding organisms from photoinhibition or light-induced damage [42]. A sharp rise in NPQ was observed in both ND and NR treatments from day 0 to day 1, peaking on day 1 and declining thereafter. Notably, the NPQ levels in the ND group were significantly elevated relative to the NR group on days 1 and 5, while no significant difference was detected on day 10 (Figure 3C). This result aligns with the findings of Scibilia [43]. Additionally, under a light intensity of 200 μmol m^2^ s^−1^, no significant disparities in the relative electron transport rate (rETR) were noted between the two groups on days 1, 5, and 10 (Figure 3D–F). However, on days 1 and 5, the rETR-max in the NR group was significantly elevated compared to the ND group (Figure 3D,E). On day 10, the rETR-max values were comparable between the two groups (Figure 3F). These results suggest that, under high light conditions, ND predominantly affects NPQ and rETR-max in *H. pluvialis* during the early to mid-induction phase, while algal cells progressively adapt to environments with ND over time.

### 3.4. Effects of Nitrogen Deprivation on Contents of Total Sugar, Protein, Starch, and Lipid

**Figure 4:**
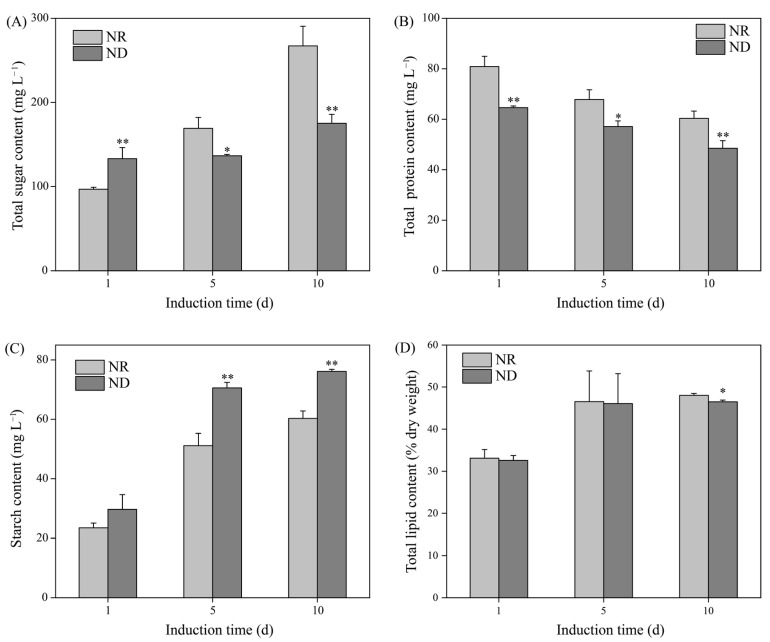
Comparison of total sugar (**A**), protein (**B**), starch (**C**), and lipid (**D**) contents between NR and ND groups. * and ** indicate significant (*p* < 0.05) and highly significant (*p* < 0.01) differences, respectively, between the NR and ND groups for the corresponding index at the same time point.

Under high light conditions, the total sugar content increased progressively in both the ND and NR groups throughout the induction period (Figure 4A). On day 1, the ND group exhibited a significantly higher sugar concentration (133.13 mg L^−1^) than the NR group (96.75 mg L^−1^). However, the total sugar levels in the ND group (136.72 and 175.30 mg L^−1^) reduced by 19.52% and 34.40% on days 5 and 10, respectively, in comparison with the NR group (Figure 4A). The protein content in both the ND and NR groups gradually decreased over the induction period, with the ND group consistently exhibiting significantly lower protein levels. On days 1, 5, and 10, the total protein content in the ND group was 64.60 mg L^−1^, 57.11 mg L^−1^, and 48.52 mg L^−1^, respectively, which was 20%, 15%, and 20% lower than the content in the NR group (Figure 4B).

In addition, the starch content in both groups increased with the duration of the induction. Notably, the ND group exhibited increases of 38.13% (70.57 mg L^−1^) and 26.21% (76.08 mg L^−1^) in starch content on days 5 and 10, respectively, relative to the NR group (Figure 4C). Similarly, the lipid content in both groups also increased over time. No significant differences were observed between the ND and NR groups on days 1 and 5. On day 10, the lipid content in the ND group (46.50% of dry weight) was slightly lower than that in the NR group (48.05% of dry weight) (Figure 4D). In summary, under high light conditions, ND suppressed the accumulation of total sugars and proteins in *H. pluvialis* while promoting starch accumulation, with minimal impact on the lipid content.

### 3.5. Effect of Nitrogen-Deprivation on Free Fatty Acid Contents

**Figure 5:**
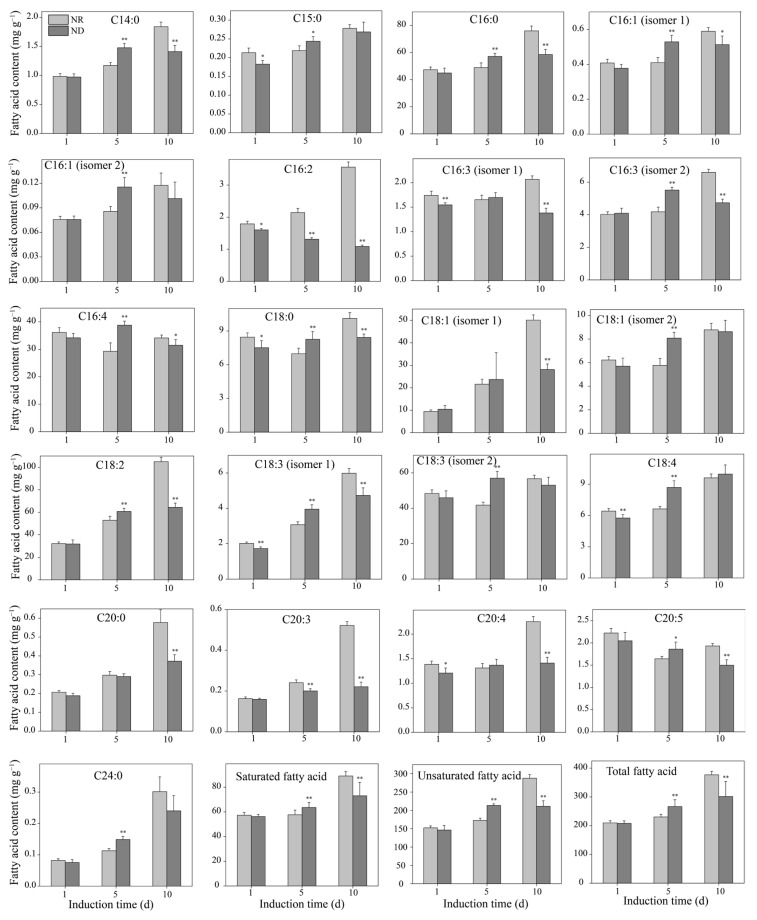
Comparison of free fatty acid contents between NR and ND groups. * and ** indicate significant (*p* < 0.05) and highly significant (*p* < 0.01) differences, respectively, between the NR and ND groups for the corresponding index at the same time point.

In *H. pluvialis*, over 95% of astaxanthin is present as fatty acid esters, primarily in the form of monoesters bound to fatty acids. These esters are stored within lipid droplets, which are predominantly composed of triglycerides [44,45]. Therefore, the dynamic changes in free fatty acid (FFA) content during the induction period were further investigated. The contents of most FFAs in both the ND and NR groups increased with the prolongation of the high light exposure (Figure 5). On day 1, there were no significant differences in the levels of saturated fatty acids, unsaturated fatty acids, or total fatty acids between the two groups (Figure 5). However, on day 5, the ND group showed significantly higher levels of saturated, unsaturated, and total fatty acids compared to the NR group, with the results on day 10 being the complete opposite. Specifically, on day 5, among the 21 detected fatty acids, 15 FFAs were remarkably elevated in the ND group, namely, C_14:0_, C_15:0,_ C_16:0_, C_16:1_ (isomer 1), C_16:1_ (isomer 2), C_16:3_ (isomer 2), C_16:4_, C_18:0_, C_18:1_ (isomer 2), C_18:2_, C_18:3_ (isomer 1), C_18:3_ (isomer 2), C_18:4_, C_20:5_, and C_24:0_, while two fatty acids (C_16:2_ and C_20:3_) were significantly decreased. The carbon chain lengths of fatty acids involved in astaxanthin esterification in *H. pluvialis* mainly range from 16 to 18 carbons. Conversely, on day 10, the ND group exhibited significantly lower levels of 15 FFAs compared to the NR group, namely, C_14:0_, C_16:0_, C_16:1_ (isomer 1), C_16:2_, C_16:3_ (isomer 1), C_16:3_ (isomer 2), C_16:4_, C_18:0_, C_18:1_ (isomer 1), C_18:2_, C_18:3_ (isomer 1), C_20:0_, C_20:3_, C_20:4_, and C_20:5_. This shift is almost the opposite of what was observed on day 5, suggesting a dynamic alteration in fatty acid metabolism over time.

### 3.6. Comparative Transcriptome Analysis

#### 3.6.1. Overview of the Transcriptomic Difference

Transcriptomic analyses of *H. pluvialis* cells under ND and NR conditions were performed using RNA-Seq on day 1 and 5 of high light exposure. On average, 21.43 million clean reads were obtained per sample, and a total of 32,204 genes were detected. Additionally, the sequencing saturation curves (Figure S1) indicate that the gene detection had reached a plateau, confirming that the sequencing depth was sufficient. In our previous study, the transcriptomic profiles of both ND and NR groups on day 5 were analyzed, primarily focusing on the analysis of the relationship between pyrimidine metabolism and SCW synthesis in *H. pluvialis*, aiming to identify methods to enhance the yield of astaxanthin-rich motile cells [19]. However, that study did not elucidate the impact of ND on other critical metabolic pathways in *H. pluvialis* on day 5. To gain a more comprehensive understanding of the effects of ND on the transcriptome during the early and middle stages of cultivation, transcriptomic data on both days 1 and 5 were used in this study. In addition, to assess the accuracy and reproducibility of the RNA-seq results, six randomly selected genes (two from the astaxanthin biosynthesis pathway, two from the lipid biosynthesis pathway, and two related to carbon–nitrogen metabolism pathways) were validated using qRT-PCR. The expression trends were consistent with the transcriptome data (Figure S2), thereby confirming the reliability of the sequencing results.

**Figure 6:**
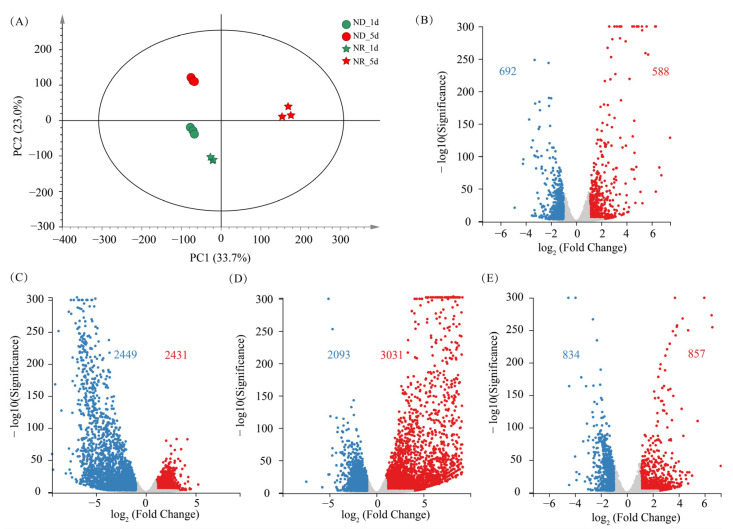
Overview of transcriptomic changes in *H. pluvialis*. (**A**) Principal component analysis (PCA) score plot of transcriptomes. (**B**) Volcano plots of differentially expressed genes (DEGs) between NR and ND groups on day 1. (**C**) Volcano plots of DEGs between NR and ND groups on day 5. (**D**) Volcano plots of DEGs between days 1 and 5 in the NR group. (**E**) Volcano plots of DEGs between days 1 and 5 in the ND group. Red numbers indicate up-regulated DEGs count in ND group relative to NR group; blue numbers indicate down-regulated DEGs count.

To obtain a global view of the effects of ND and culture time on the transcriptome of *H. pluvialis*, the RNA-seq data were subjected to principal component analysis (PCA). Samples from all the groups are distinctly separated in the PCA score plot (Figure 6A), indicating that both ND and culture time significantly affect the transcriptome of algal cells. Three biological replicates within the same group clustered tightly together, suggesting minimal intra-group variation, which implies that the transcriptomic results are stable and reliable.

On day 1, a total of 1280 differentially expressed genes (DEGs) were identified between the ND and NR groups, comprising 692 upregulated expression genes (URGs) and 588 downregulated expression genes (DRGs). On day 5, the number of DEGs had markedly increased to 4880, with 2431 URGs and 2449 DRGs. In contrast, the transcriptomic differences between day 1 and day 5 within the ND group yielded only 1691 DEGs, substantially fewer than the 5124 DEGs detected in the NR group. This observation corresponds with the more evident morphological alterations in NR group cells (Figure 1). While the ND group primarily showed astaxanthin accumulation without notable structural changes, cells in the NR group exhibited significant morphological transformation over the same period. These findings suggest a positive association between DEG abundance and the extent of cellular morphological variation, with more dramatic transcriptomic shifts accompanying pronounced phenotypic changes.

#### 3.6.2. GO and KEGG Pathway Enrichment Analysis

**Figure 7:**
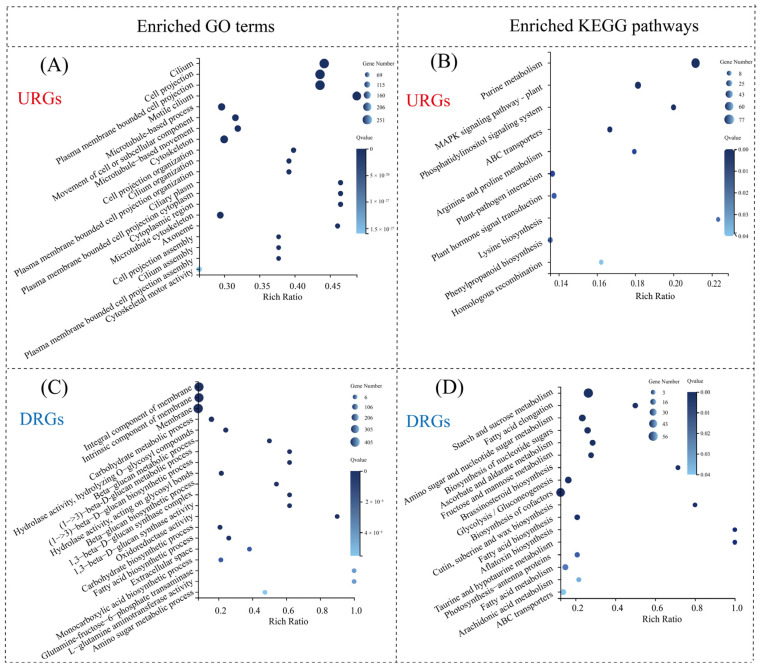
Overview of enriched GO terms and KEGG pathways among DEGs between NR and ND groups on day 1. (**A**) Enriched KEGG pathways of URGs; (**B**) enriched GO terms of URGs; (**C**) enriched KEGG pathways of DRGs; (**D**) enriched GO terms of DRGs.

To further elucidate the regulatory mechanisms by which *H. pluvialis* responds to ND, GO and KEGG pathway enrichment analyses of the DEGs were performed. The URGs on day 1 were significantly enriched in seven KEGG metabolic pathways, including alanine, aspartate, and glutamate metabolism; nitrogen metabolism; and arginine biosynthesis (Figure 7A). Additionally, the GO enrichment analysis revealed that the URGs on day 1 were significantly enriched in 75 “biological processes”, 56 “molecular functions”, and five “cellular components”, with the top three enrichments related to the biosynthesis and metabolic processes of glutamine family amino acids and arginine biosynthesis (Figure 7B). Both GO and KEGG analyses indicated that the URGs on day 1 were primarily associated with amino acid metabolism and biosynthesis, in sharp contrast to the findings on day 5. On day 5, the enriched KEGG pathways for URGs were purine metabolism, the MAPK signaling pathway, and the phosphatidylinositol signaling system, and the enriched GO terms for URGs were related to flagella, cell projections, and plasma membrane-bound projections [19].

On day 1, the DRGs were significantly enriched in eight KEGG pathways, with the top three being cysteine and methionine metabolism, arachidonic acid metabolism, and glutathione metabolism (Figure 7C). The GO analysis further revealed significant enrichment in 178 terms, including 100 “biological processes”, 25 “cellular components”, and 53 “molecular functions”. The categories most enriched included cellular amino acid metabolism, organic acid metabolism, and oxoacid metabolic processes (Figure 7D). Notably, both KEGG and GO enrichment patterns for DRGs differed markedly from those observed on day 5. At this later stage, the enriched GO terms were predominantly associated with membrane composition, while the top KEGG pathways included starch and sucrose metabolism, fatty acid elongation, and amino sugar and nucleotide sugar metabolism [19]. These findings suggest distinct functional roles of DRGs between the early and middle stages of nitrogen deprivation, indicating that *H. pluvialis* exhibits stage-specific transcriptomic responses under high light conditions.

#### 3.6.3. Impact of Nitrogen Deprivation on Astaxanthin Synthesis Pathway in *H. pluvialis*

**Figure 8:**
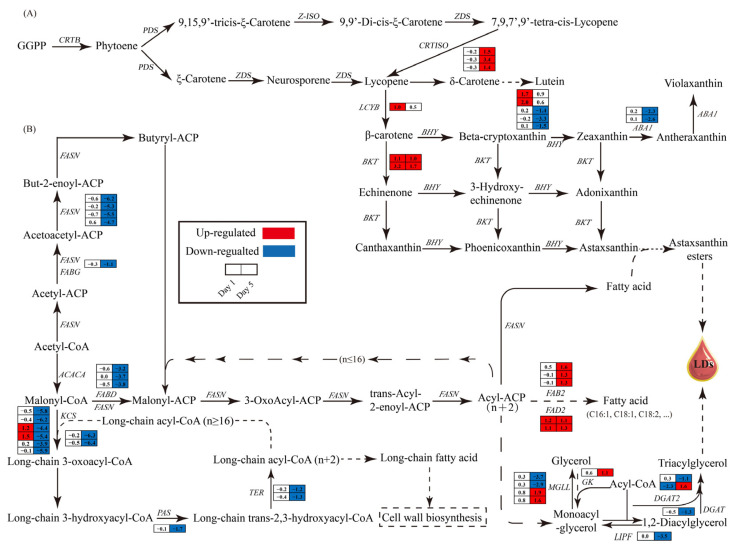
Transcriptomic comparison of astaxanthin biosynthesis pathway (**A**) and fatty acid and glycerolipid biosynthesis pathway (**B**) between NR and ND groups. Log_2_ fold changes in gene expression between ND and NR conditions are presented beside each gene. Solid arrows indicate single-step reactions, while dashed arrows indicate multi-step reactions. Red of the cells indicates genes up-regulated in the ND group compared with the NR group, while blue indicates down-regulated genes. The full names of the enzyme coding genes were listed in Table S5.

Given that ND enhances astaxanthin accumulation in *H. pluvialis* cells under high light conditions, it is imperative to explore the transcriptional alterations in genes associated with the astaxanthin biosynthesis pathway. On day 1, genes encoding lycopene cyclase (LCYB), beta-carotene ketolase (BKT), and beta-carotene hydroxylase (BHY), which are key enzymes for astaxanthin biosynthesis, were significantly upregulated in the ND group compared to the NR group (Figure 8A). On day 5, the expression of lycopene isomerase (CRTISO) and *BKT* was also increased in the ND treatment (Figure 8A). These transcriptional shifts are consistent with previous studies reporting ND-induced upregulation of core astaxanthin biosynthesis genes in *H. pluvialis* [32,46]. Furthermore, on day 5, three *BHYs* and two zeaxanthin epoxidase (ABA1) genes were markedly downregulated in the ND group (Figure 8A).

#### 3.6.4. Impact of Nitrogen Deprivation on Lipid Synthesis Pathway

In *H. pluvialis*, astaxanthin is predominantly accumulated as fatty acid esters within lipid droplets enriched in triglycerides [6]. Hence, the impact of ND on the expression of genes in the lipid synthesis pathway was analyzed, focusing on the fatty acid synthesis and triglyceride synthesis pathways. On day 1, there were no significant differences in the expression levels of most genes between the ND and NR groups (Figure 8B), which is consistent with the free fatty acid content measurements (Figure 5). However, on day 5, the genes encoding key enzymes in the fatty acid synthesis pathway, including acetyl-coA carboxylase (ACACA), fatty acid synthase (FASN), 3-oxoacyl-[acyl-carrier protein] reductase (FABG), and acyl carrier protein s-malonyltransferase (FABD), were markedly downregulated in the ND group relative to the NR group (Figure 8B). This outcome contrasts with the alterations in free fatty acid levels observed on day 5 but aligns with the changes noted on day 10 (Figure 5), indicating that the alterations in gene expression occur prior to the variations in fatty acid levels.

Moreover, the expression levels of genes involved in synthesizing very-long-chain fatty acids (VLCFAs), including those encoding 3-ketoacyl-coA synthase (KCS), very long chain (3R)-3-hydroxyacyl-coA dehydratase (PAS), and very long-chain enoyl-coA reductase (TER), were significantly downregulated in the ND group (Figure 8B), indicating that the synthesis of VLCFAs may be inhibited in the ND group. VLCFAs are important components of the SCW of *H. pluvialis* [20]. The downregulation of these genes is consistent with the absence of an SCW in cells in the ND group. In contrast, genes encoding key enzymes for unsaturated fatty acid synthesis, such as Δ6-fatty acid desaturase (FAD2) and acyl-[acyl-carrier protein] desaturase (FAB2), were significantly upregulated in the ND group compared to the NR group (Figure 8B), which is consistent with the changes in unsaturated fatty acid contents on day 5, as presented in Figure 5.

On day 5, the expression levels of four monoglyceride lipase (MGLL) genes and two diacylglycerol o-acyltransferase 2 (DGAT2) genes exhibited significant variability, with half upregulated and half downregulated in the ND group. Notably, *DGAT2* transcription was reduced by 79.59% on day 1. Additionally, the gene encoding glycerol kinase (GK) was upregulated by 1.1 times on day 5, while triacylglycerol lipase (LIPF) and diacylglycerol acyltransferase (DGAT) were downregulated by 91.15% and 60.00%, respectively. In summary, ND significantly affected the transcription levels of enzyme-encoding genes involved in the lipid biosynthesis pathways of *Haematococcus pluvialis*.

#### 3.6.5. Impact of Nitrogen Deprivation on Expression of Photosynthesis-Related Genes

**Figure 9:**
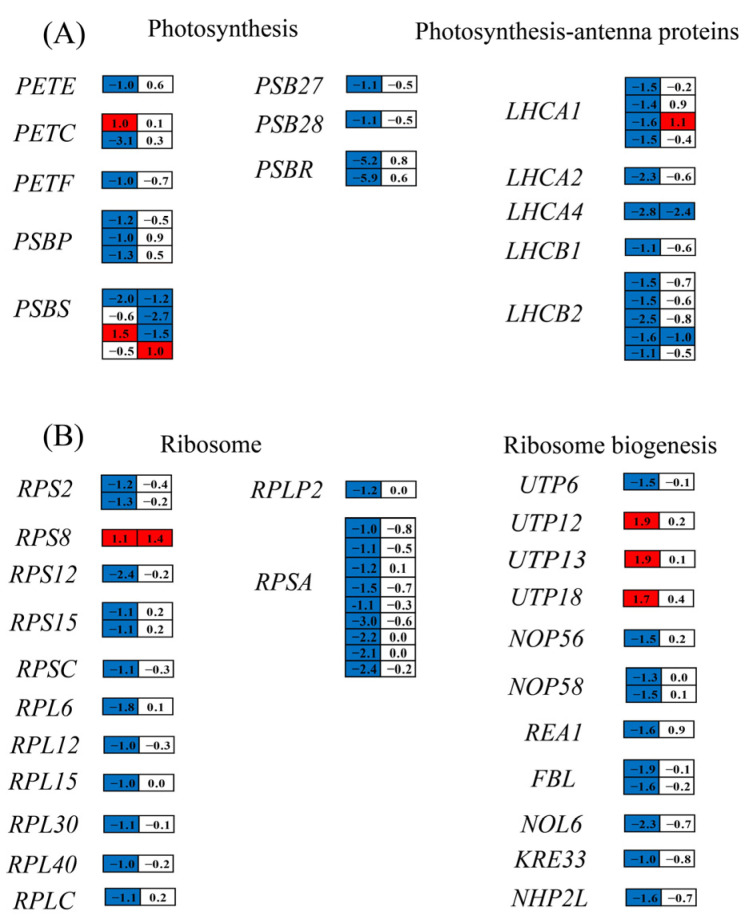
DEGs associated with photosynthesis (**A**) and ribosome biogenesis (**B**) in the NR and ND groups. Red of the cells indicates genes up-regulated in the ND group compared with the NR group, while blue indicates down-regulated genes. The full names of the enzyme coding genes were listed in Table S6.

ND influenced the chlorophyll fluorescence parameters of *H. pluvialis* (Figure 3), prompting an analysis of its impact on the transcription of photosynthesis-related genes. On day 1, the ND group exhibited significant downregulation of genes encoding plastocyanin (PETE), ferredoxin (PETF), oxygen-evolving enhancer protein 2 of photosystem II (PSBP), PSBR, PSB27, PSB28, LHCA1, LHCA2, LHCA4, LHCB1, and LHCB2, compared to the NR group (Figure 9A). In contrast, genes encoding PETC and PSBS displayed variable expression patterns, with both upregulation and downregulation observed under ND conditions on the same day. On day 5, only a few photosynthesis-related genes displayed significantly differential expression levels between the ND and NR groups, and most of them were downregulated in the ND group. The downregulation of photosynthesis-related genes in the ND group was consistent with the lower chlorophyll fluorescence parameters observed in the ND group, as presented in Figure 3.

#### 3.6.6. Impact of Nitrogen Deprivation on Sugar Metabolism Pathway

**Figure 10:**
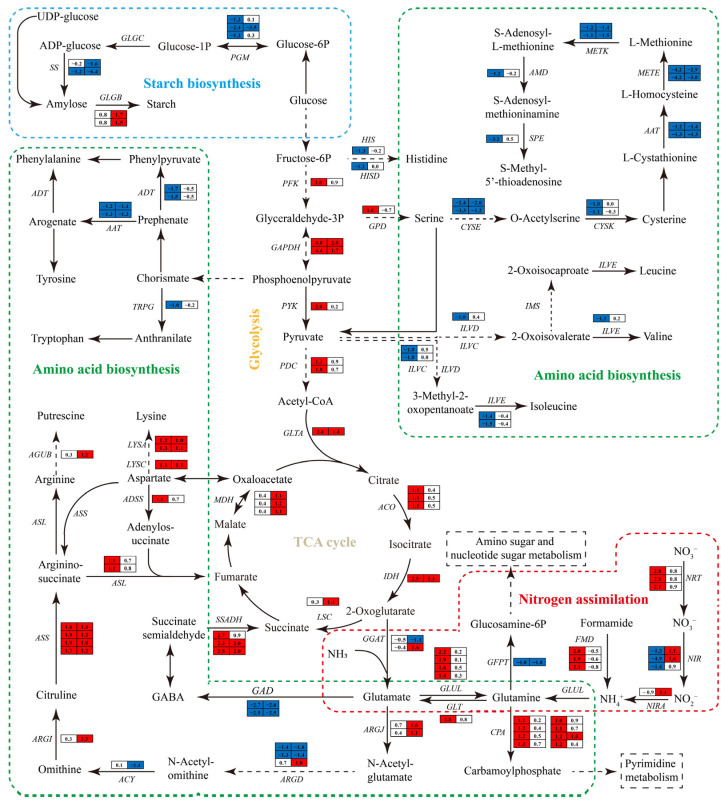
DEGs involved in sugar metabolism pathway and amino acid biosynthesis pathway. Solid lines indicate single-step reactions, dashed lines indicate multi-step reactions. Red of the cells indicates genes up-regulated in the ND group compared with the NR group, while blue indicates down-regulated genes. The full names of the enzyme coding genes were listed in Table S7.

On day 1, genes involved in the glycolysis pathways, such as 6-phosphofructokinase (PFK), pyruvate kinase (PYK), glyceraldehyde 3-phosphate dehydrogenase (GAPDH), and pyruvate decarboxylase (PDC), were markedly upregulated in ND group relative to the NR group (Figure 10). Most of these genes maintained elevated expression levels in the ND group on day 5. In the tricarboxylic acid (TCA) cycle, the gene transcript levels encoding aconitate hydratase (ACO) and isocitrate dehydrogenase (IDH) were notably upregulated on day 1, while those encoding IDH, succinyl-coA synthetase (LSC), and malate dehydrogenase (MDH) were markedly upregulated on day 5.

Additionally, in the starch biosynthesis, the transcript levels of the genes encoding phosphoglucomutase (PGM) and starch synthase (SS) were markedly downregulated in the ND group compared to the NR group. On day 1, the expression levels of these genes decreased by 59.39–81.05% and 56.47%, respectively, and further declined by 91.16% and 67.01–98.81% on day 5. In contrast, the gene encoding 1,4-α-glucan branching enzyme (GLGB) was significantly upregulated on day 5, with transcript levels that were increased by 1.83–2.24-fold relative to the NR group. Collectively, these transcriptional changes suggest that *H. pluvialis* reprograms its energy and carbohydrate metabolism in response to nitrogen deprivation, reflecting a coordinated adaptation to nutrient stress.

#### 3.6.7. Impact of Nitrogen Deprivation on Nitrogen Metabolism Pathways

In the nitrogen uptake and assimilation pathway (Figure 10), the expression levels of genes encoding nitrate/nitrite transporter (NRT), formamidase (FMD), glutamine synthetase (GLUL), carbamoyl phosphate synthase (CPA), and glutamate synthase (GLT) were markedly elevated in the ND group compared to the NR group on day 1, with fold increases of 3.29–5.96, 2.73–3.29, 1.64–3.59, 1.00–1.46, and 5.96, respectively. In contrast, the transcripts of nitrate reductase (NIR), glutamine-fructose-6-phosphate transaminase (GFPT), and glutamate decarboxylase (GAD) were markedly downregulated, by 59.39–96.65%, 50%, and 82.32–84.61%, respectively. On day 5, the ND group exhibited significant upregulation of *NIR*, *NIRA*, *CPA*, and *ARGJ*, whereas *GFPT* and *GAD* remained significantly suppressed compared to the NR group. Notably, NR expression was significantly reduced in the ND group on day 1, while FMD expression was markedly increased.

In the amino acid biosynthesis pathway, most of the DEGs related to amino acid biosynthesis were observed on day 1, with the majority of them significantly downregulated in the ND group compared to the NR group. These included, for instance, genes encoding enzymes involved in the biosynthesis of branched-chain amino acids (i.e., isoleucine, valine, and leucine), including branched-chain amino acid aminotransferase (ILVE), dihydroxy-acid dehydratase (ILVD), and ketol-acid reductoisomerase (ILVC). Similarly, genes related to the aromatic amino acid pathways (i.e., tyrosine, phenylalanine, tryptophan), including anthranilate synthase (AS), arogenate/prephenate dehydratase (ADT), and aspartate prephenate aminotransferase (AAT), were significantly downregulated in the ND group. In addition, the transcript levels of genes associated with histidine and methionine synthesis, including imidazoleglycerol phosphate synthase (HIS), serine O-acetyltransferase (CYSE), cysteine synthase (CYSK), 5-methyltetrahydropteroyltriglutamate homocysteine methyltransferase (MEME), S-adenosylmethionine synthetase (METK), S-adenosylmethionine decarboxylase (AMD), and spermidine synthase (SPE), were markedly reduced in the ND group on day 1.

In contrast, genes involved in lysine synthesis, including aspartate kinase (LYSC) and diaminopimelate decarboxylase (LYSA), as well as those participating in putrescine synthesis, including ornithine carbamoyltransferase (ARG1), argininosuccinate synthase (ASS), argininosuccinate lyase (ASL), and N-carbamoyl putrescine amidase (AGUB), showed a significant increase in expression on either day 1 or 5 under ND conditions. Specifically, *LYSA* expression increased by 2.30–2.46-fold and 2.00–2.14-fold on days 1 and 5, respectively; *ADSS* and *ASL* were upregulated by approximately 1.14-fold on day 1; and *ARGI*, *ASS*, and *AGUB* were upregulated by 3.92-fold, 2.03–2.73-fold, and 1.29-fold, respectively, on day 5. In summary, nitrogen deprivation induced substantial transcriptional changes in *H. pluvialis*, particularly in genes associated with nitrogen uptake, assimilation, and amino acid biosynthesis. These changes, including both upregulation and downregulation of key genes at different time points, reflect the organism’s metabolic adaptation to nitrogen-limited conditions.

#### 3.6.8. Impact of Nitrogen Deprivation on Ribosome Biogenesis and Ubiquitin–Proteasome Degradation Pathway

The notable decrease in protein content observed in the ND group relative to the NR group (Figure 4B) allowed us to examine the DEGs involved in protein synthesis and degradation pathways. Ribosomes, large ribonucleoprotein complexes composed of ribosomal RNA and ribosomal proteins, serve as the protein factories of the cell [47]. The transcript levels of 12 ribosomal protein genes (RPS2, RPS12, RPS15, RPSC, RPL6, RPL12, RPL15, RPL30, RPL40, RPLC, RPLP2, RPSA) were markedly reduced in the ND group relative to the NR group on day 1. Furthermore, the expression levels of eight genes involved in the ribosome biogenesis process were also significantly downregulated in the ND group. On day 5, there were no significant differences in gene expression related to ribosomal components and ribosome biogenesis between the ND and NR groups (Figure 9B). However, the protein content in the ND group remained markedly lower than that in the NR group on days 5 and 10 (Figure 4B), implying that additional pathways may be involved in regulating protein levels.

The ubiquitin proteasome system (UPS) is the primary pathway for protein degradation in eukaryotic cells [48,49]. As detailed in Supplementary Table S2, the effects of ND on the expression of genes involved in the UPS were primarily observed on day 5. In the ND group, the expression levels of three genes encoding ubiquitin were significantly altered on day 5, including one upregulated and two downregulated genes. The expression level of a gene encoding a HECT-type E3 ubiquitin ligase was significantly downregulated, while the expression levels of one gene encoding U-box type E3, five genes encoding single RING-finger type E3, and six genes encoding multi-subunit RING-finger type E3 were all significantly upregulated in the ND group compared to the NR group. E3 ubiquitin ligases are crucial in the ubiquitination pathway as they recognize specific substrate proteins and promote the ubiquitination process, thereby marking proteins for degradation [50]. The expression levels of most types of E3 ubiquitin ligases were markedly elevated in the ND group compared to the NR group, potentially enhancing protein degradation and thereby contributing to the reduced protein content observed under ND conditions (Figure 4B).

#### 3.6.9. Impact of Nitrogen Deprivation on Cell Wall Biosynthesis-Related Genes

**Figure 11:**
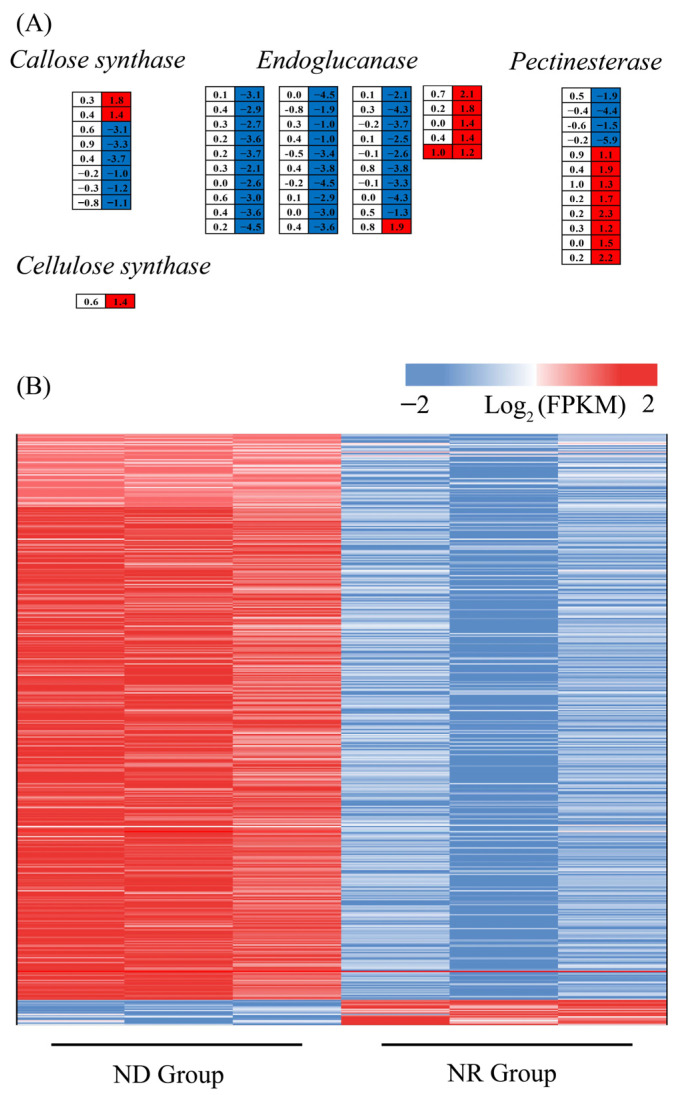
DEGs associated with cell wall biosynthesis (**A**) and cell movement (**B**). Red of the cells indicates genes up-regulated in the ND group compared with the NR group, while blue indicates down-regulated genes.

As ND suppressed SCW biosynthesis in *H. pluvialis* (Figure 1), the expression profiles of cell wall biosynthesis genes (CWBGs) were subsequently investigated. The CWBGs showed minimal differential expression between the ND and NR groups on day 1, while a significant number were differentially expressed on day 5. The expression levels of 36 genes encoding endoglucanase, eight genes encoding callose synthase, 12 genes encoding pectinesterase, and one gene encoding cellulose synthase were significantly altered on day 5. Among the genes examined, there were notable decreases in the expression levels of 30 endoglucanase genes, six callose synthase genes, and three pectinesterase genes in the ND group compared to the NR group (Figure 11A). Furthermore, on day 1, no marked difference was observed between the NR and ND groups in the transcription of genes associated with mannan synthesis. On day 5, however, several key genes in this pathway—including mannose-1-phosphate guanylyltransferase (GMPP) gene, the alpha 1,6-mannosyltransferase (OCH1) gene, mannan synthase (CSLA) gene, and eight mannosyltransferase (MNNs) genes—exhibited markedly reduced transcription levels in the ND group [19]. Cellulose, glucose, fructose, and mannose are critical components of the polysaccharides that constitute the cell walls of *H. pluvialis*, with cellulose being the primary component of the primary cell wall and mannose predominant in the secondary cell wall [51,52]. Collectively, these findings suggest that ND may inhibit SCW biosynthesis in *H. pluvialis* primarily by suppressing the transcription of CWBGs.

#### 3.6.10. Impact of Nitrogen Deprivation on Movement-Related Genes in *H. pluvialis*

On day 1, the majority of the motility genes in both the ND and NR groups displayed comparable transcription levels, as cells in both groups possessed flagella and exhibited motility at this timepoint. On day 5, however, the flagella of the algae in the NR group had detached, while those in the ND group remained intact. This resulted in the differential expression of 469 out of the 945 genes associated with cell movement between the two groups. Specifically, a total of 449 genes were significantly upregulated, while 20 genes were markedly downregulated in the ND group relative to the NR group (Figure 11B). The upregulated genes included those encoding ADP-ribosylation factor (ARF) proteins [53], intraflagellar transport (IFTs) proteins, and kinesin family member (KFM), all of which play critical roles in flagellar movement and assembly [54,55]. In contrast, the downregulated genes primarily encoded aurora protein kinases, which are important for regulating flagellar detachment and disassembly [56]. The downregulation of these kinases in the ND group aligns with the observed retention of flagella and the maintenance of motility in these cells.

#### 3.6.11. Impact of Nitrogen Deprivation on ROS Scavenging-Related Genes in *H. pluvialis*

Stress conditions trigger the activation of both enzymatic and non-enzymatic antioxidant defense systems in *H. pluvialis*, functioning synergistically to neutralize the accumulation of ROS and restore intracellular redox homeostasis [57,58]. As shown in Figure 2, on day 1, the transcript levels of ferritin and glutathione reductase (GR) in the ND group were significantly upregulated. Conversely, genes encoding superoxide dismutase (SOD) and peroxiredoxin (PRXR) were downregulated. Among the six differentially expressed thioredoxin (TRX) genes, five were downregulated and one was upregulated in the ND group. On day 5, the transcript levels of thioredoxin reductase (TRXR), TRX, and glutathione peroxidase (GPX) were significantly upregulated in the ND group relative to the NR condition. Conversely, genes encoding SOD, ferritin, and GR showed reduced expression in the ND group. Both the downregulation and upregulation of genes encoding PRXR in the ND group were observed on day 5 (Supplementary Table S3). These findings indicated that *H. pluvialis* differentially regulated the expression of ROS-scavenging enzyme genes to maintain ROS homeostasis in response to ND under high light conditions.

## 4. Discussion and Conclusions

*H. pluvialis* typically exhibits coordinated activation of secondary cell wall (SCW) formation and astaxanthin accumulation under stress. However, under nitrogen deprivation (ND), it displays a distinct metabolic preference by prioritizing astaxanthin biosynthesis while suppressing SCW formation. To elucidate this phenomenon, we analyzed the physiological and transcriptional responses of *H. pluvialis* under ND conditions.

Astaxanthin biosynthesis represents a key adaptive response to ND stress. Following ND induction, genes encoding critical enzymes in the astaxanthin biosynthetic pathway (e.g., *LCYB*, *BKT*, *BHY* and *CRTISO*) were rapidly upregulated. This establishes the molecular basis for accelerated astaxanthin production by ensuring precursor availability [59]. Notably, ND markedly suppressed the expression of zeaxanthin epoxidase (*ABA1*). Since ABA1 converts zeaxanthin to violaxanthin—diverting flux away from astaxanthin (via *BKT*)—its downregulation likely favors astaxanthin accumulation [60]. Thus, ND enhances astaxanthin biosynthesis both by upregulating core pathway genes and by suppressing competing metabolic routes. While astaxanthin and lipid accumulation are often correlated in *H. pluvialis* [6,29], it is important to emphasize that in the present study, the transcript levels of key lipid biosynthesis genes (*DGAT* and *DGAT2*) [61] were significantly downregulated under ND conditions. This downregulation likely drives the observed reduction in lipid content, indicating that the astaxanthin and lipid biosynthesis pathways are not strictly coupled. This decoupling may reflect fundamentally distinct synthetic pathways and the substantially higher cellular lipid baseline compared to astaxanthin.

Nitrogen stress universally suppresses photosynthetic activity [25]. Consistently, ND downregulated photosynthesis-related genes (Figure 9), impairing photosynthetic performance (Figure 3). This inhibition disrupts the cellular energy balance as reduced energy consumption leads to the accumulation of excess excitation energy. Overexcited chlorophyll reacts with O_2_, generating singlet oxygen (^1^O_2_) [62]. Concurrently, impaired electron transport promotes electron leakage to O_2_, forming superoxide anions (O_2_^−^) [63]. Consequently, suppressed photosynthesis is the primary driver of the rapid ROS burst observed early in ND treatment. This ROS surge subsequently activates enzymatic and non-enzymatic antioxidant defenses to restore redox homeostasis [57,58]. Accordingly, 31 ROS-scavenging genes were differentially regulated on days 1 or 5, modulating antioxidant enzyme/compound synthesis and intracellular ROS levels (Figure 2).

Glycolysis, the TCA cycle, and amino acid metabolism are central hubs of carbon/nitrogen metabolism and energy provision [64,65]. TCA cycle intermediates supply essential carbon skeletons for compounds like carotenoids and lipids. Exogenous supplementation with glycolysis/TCA intermediates enhances astaxanthin accumulation in *H. pluvialis* [10,11,66,67,68]. Notably, on day 1 post-ND, key glycolysis (e.g., *PFK*, *GAPDH*, *PYK*) and TCA cycle (e.g., *GLTA*, *ACO*, *IDH*) genes were significantly upregulated. This indicates ND activation of both pathways, providing increased energy and precursors for astaxanthin biosynthesis. In addition, ammonium (NH_4_^+^) and nitrate (NO_3_^−^) are primary inorganic nitrogen sources for algae. NH_4_^+^ is assimilated directly via transporters or derived from formamide by formamidase (FMD), and then converted to glutamine/glutamate by glutamine synthetase (GLUL) and glutamate synthase (GLT). NO_3_^−^ is imported via nitrate transporters (NRT), reduced to NH_4_^+^ by nitrate reductase (NIR) and nitrite reductase (NIRA), and assimilated [69]. In this study, ND upregulated *GLUL* and *GLT* expression, suggesting enhanced nitrogen assimilation capacity. Strikingly, NIR was downregulated while *FMD* was upregulated on day 1, indicating a shift toward utilizing intracellular reserves (e.g., formamide-derived NH_4_^+^) under nitrogen scarcity. Glutamine metabolism diverges via GFPT (to glucosamine-6-phosphate for amino sugar/nucleotide synthesis) or CPA (to carbamoyl phosphate for pyrimidine biosynthesis). ND downregulated *GFPT* but upregulated *CPA*, demonstrating nitrogen allocation reprogramming. Lysine accumulation correlates positively with glycolysis consistently [70], and our data also show concurrent upregulation of lysine biosynthetic genes and most of the genes involved in glycolysis under ND conditions. ND also activated putrescine biosynthesis genes (*ARG1*, *ASS*, *ASL*, *AGUB*), potentially increasing putrescine production—a known promoter of astaxanthin accumulation [71].

Beyond enhanced astaxanthin accumulation, ND-treated cells exhibited two distinctive phenotypes: absent SCW formation and retained flagella. In this study, the transcription levels of key genes involved in very-long-chain fatty acid (VLCFA) biosynthesis—such as 3-ketoacyl-CoA synthase (KCS), (3R)-3-hydroxyacyl-CoA dehydratase (PAS), and very-long-chain enoyl-CoA reductase (TER)—were significantly downregulated in ND versus NR, likely reducing VLCFA synthesis (Figure 6). As VLCFAs are essential components of the trilaminar SCW sheath, their reduced synthesis likely contributes to blocked SCW formation [20]. Furthermore, the transcription levels of most genes related to mannan synthesis, a major component of the SCW in *H. pluvialis* [13,72], were significantly downregulated in the ND group by day 5, explaining the reduced structural polysaccharide content and impaired SCW biogenesis. Conversely, over 95% of cell motility-related genes exhibited higher expression in the ND cells, enabling sustained flagellar integrity and motility.

In summary, *H. pluvialis* deploys concerted transcriptional, physiological, and morphological adaptations to cope with ND. Initial photosynthesis inhibition triggers ROS production but is rapidly counteracted by antioxidant systems. ND promotes astaxanthin and starch accumulation while suppressing total carbohydrates, protein, lipids (with mid-culture transient fatty acid increase), and SCW components. Transcriptomics reveals ND-induced upregulation of TCA cycle, glycolysis, astaxanthin biosynthesis, and motility genes, contrasted by downregulation of photosynthesis, lipid biosynthesis, ribosome biogenesis, amino acid synthesis, and cell wall synthesis genes. ROS-scavenging gene expression is also significantly modulated. This study delineates the survival strategy of *H. pluvialis* under high light ND stress from physiological and transcriptional perspectives; however, further work is required to fully elucidate the underlying metabolic regulatory networks.

## Data Availability

The data that support the findings of this study are available in the Supplementary Materials.

## Supplementary Materials

The following supporting information can be downloaded at: https://www.mdpi.com/article/10.3390/metabo15060388/s1, Figure S1: Sequencing saturation curves of different samples; Figure S2: The gene expression of six genes with randomly selected from different metabolic pathways by qRT-PCR (A) and RNA-seq (B); Table S1: The primer sequence for DEG validation by quantitative real-time PCR; Table S2: Fatty acids information and standard curves for quantitative calculations; Table S3: Impact of nitrogen-deprivation on ubiquitin-proteasome degradation pathway; Table S4: Impact of nitrogen deprivation on ROS scavenging genes in *H. pluvialis*; Table S5: The information and expression difference of astaxanthin and fatty acid fatty acid and glycerolipid biosynthesis pathway; Table S6: DEGs associated with photosynthesis and ribosome biogenesis in the NR and ND groups; Table S7: DEGs involved in sugar metabolism pathway and amino acid biosynthesis pathway; Table S8: DEGs associated with cell wall biosynthesis; Table S9: DEGs associated with cell movement on day 5.
