# Supplementary material for: Nitrogen Deprivation Drives Red Motile Cell Formation in Haematococcus pluvialis: Physiological and Transcriptomic Insights
Source: Metabolites. 2025 Jun 10;15(6):388. doi: 10.3390/metabo15060388 (PMC12195371; doi:10.3390/metabo15060388)
Supplement: Supplementary file 1 [file metabolites-15-00388-s001.zip › supplementary figures.pdf]

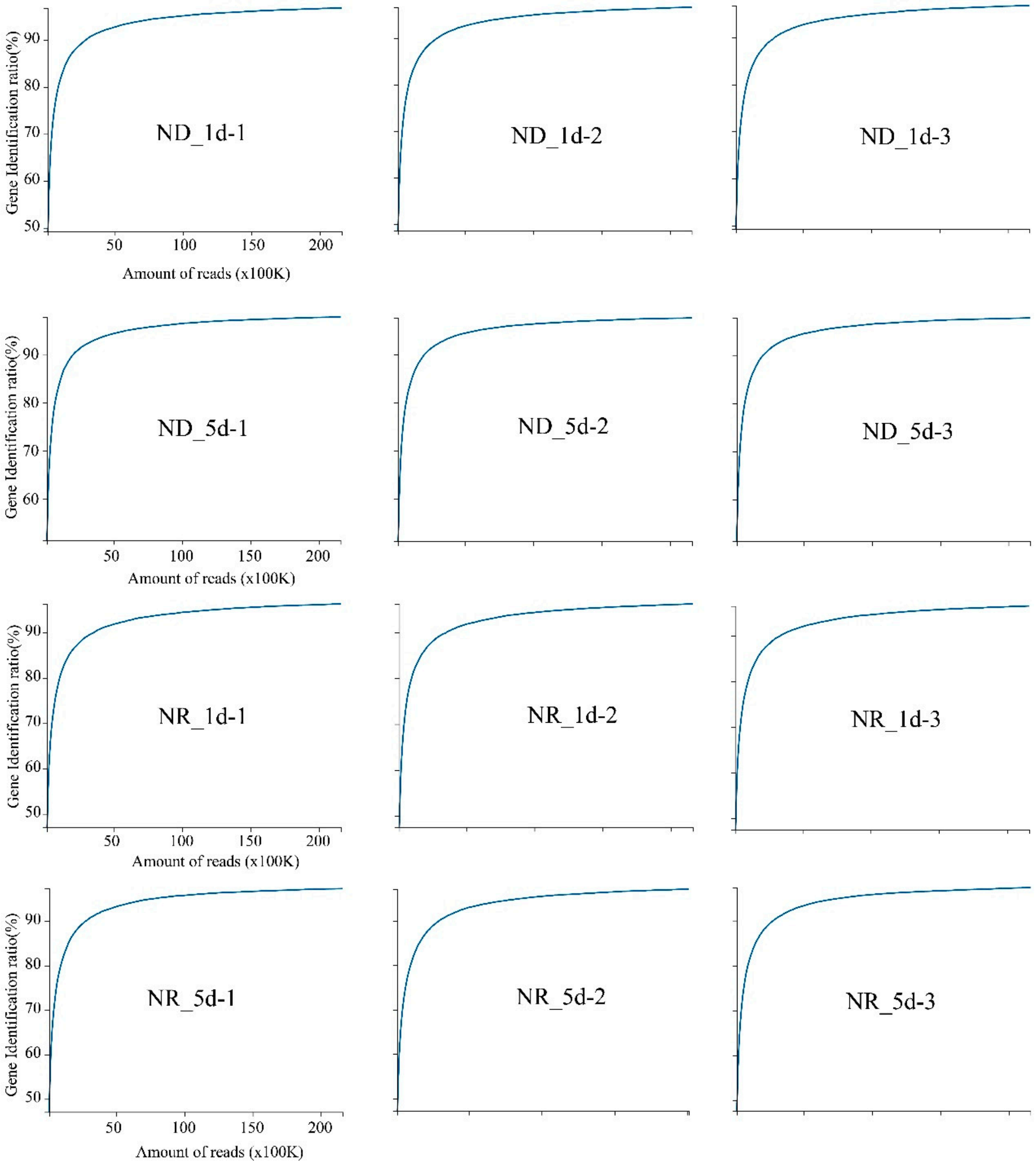

Figure S1. Sequencing saturation curves of different samples.

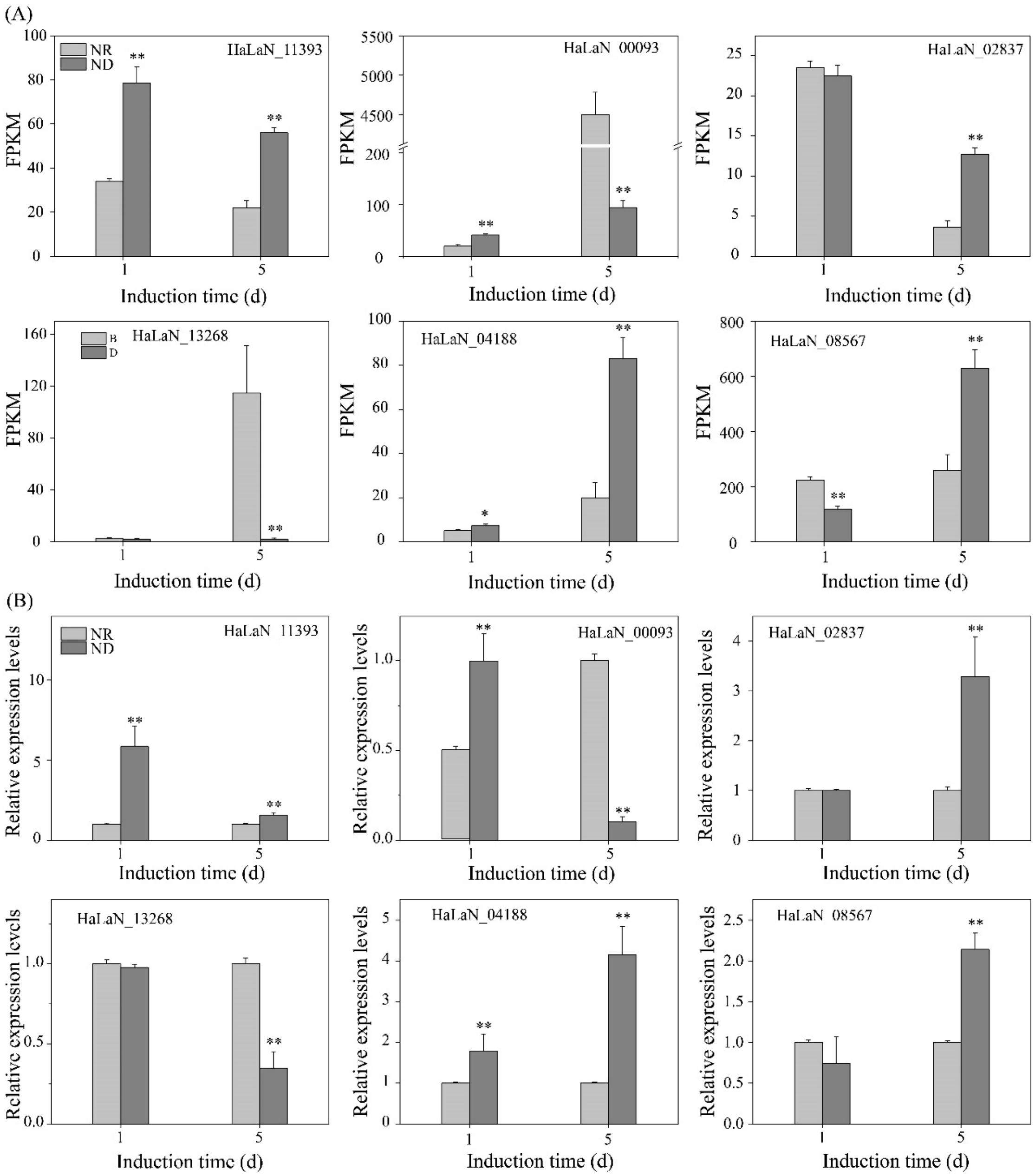

Figure S2. The gene expression of six genes with randomly selected from different metabolic pathways by RNA-seq (A) and qRT-PCR (B).
